# Somatic Genomic and Transcriptomic Changes in Single Ischemic Human Heart Cardiomyocytes

**DOI:** 10.21203/rs.3.rs-5875531/v1

**Published:** 2025-01-31

**Authors:** Nazia Hilal, Zheming An, Maksymilian Prondzynski, Erica Matsui, Debesh Sahu, Shulin Mao, Youngsook Lucy Jung, Yingxi Yang, Sonia Epstein, Ming-Hui Chen, William Pu, Federica Del Monte, August Yue Huang, Sangita Choudhury

**Affiliations:** 1 Division of Genetics and Genomics, Department of Pediatrics, Boston Children’s Hospital, Boston, MA, USA.; 2 Harvard Medical School, Boston, MA, USA.; 3 Broad Institute of MIT and Harvard, Cambridge, MA, USA.; 4 Department of Cardiology, Boston Children’s Hospital, Boston, MA, USA.; 5 Harvard Stem Cell Institute, Cambridge, MA, 02138, USA.; 6 Medical University of South Carolina, Charleston, SC, USA.

## Abstract

Heart failure is a multifaceted syndrome contributing significantly to mortality and hospitalization rates among the global population^1^. One of the prevalent causes of heart failure is ischemic heart disease (IHD), often caused by a blockage in a coronary artery, ultimately leading to the loss of myocardial tissue and contractile force^2^. The impact of this ischemic ambiance on the cardiomyocyte genome and transcriptome has not been thoroughly studied. During normal aging, cardiomyocytes progressively accumulate somatic mutations faster than many dividing cells, suggesting that internal and external factors specific to cardiomyocytes might influence this accumulation^3^. In this study, we analyzed single-cell whole-genome and transcriptome data from the left ventricle of 5 individuals with IHD and 10 healthy control individuals. We found that somatic DNA alterations significantly increase in IHD cardiomyocytes, with distinct mutational patterns indicating a disrupted DNA repair system and a cytotoxic environment, potentially associated with increased inflammatory response in the myocardium and a compensatory anti-inflammatory response in IHD. An *in vitro* iPS-derived hypoxic cardiomyocyte mutational profile indicates similar mutational spectra. Transcriptomic analysis revealed increased expression of *EGR1*, *FOS*, and collagen genes in ischemic heart cardiomyocytes, leading to a more fibrotic heart. The aberrant accumulation of DNA alterations and changes in transcriptional patterns in the ischemic heart cardiomyocytes provide insight into the development of IHD.

## Introduction

Ischemic heart disease (IHD), a multifactorial condition, is the leading cause of death worldwide. Environmental factors, genetic factors, lifestyle components, and the interplay between these components intricately influence IHD^1^. Our current understanding of how IHD develops begins with atherosclerosis, the buildup of cholesterol plaques, which can then grow and become unstable. This results in plaque rupture, thrombus formation, and subsequent blockage of the artery, leading to myocardial infarction, producing a cascade of inflammatory events and myocardial cell loss^4^. Significant advances have been made recently in identifying disease-causing genes and susceptibility genes for coronary artery disease, myocardial infarction, and ischemic stroke, but the impact of somatic mutations in IHD in human hearts has not been investigated. A large body of research has shown that atherogenesis and carcinogenesis share numerous key characteristics and several common risk factors^5^, such as obesity, age, family history, and exposure to toxins like cigarette smoking. These risk factors are known to increase the risk of cancer by causing mutations in DNA. The mechanisms by which somatic mutation leads to cancer progression have been well-documented in carcinogenesis^6–8^ but not in the case of atherogenesis and IHD. Reactive oxygen species (ROS) have been shown to induce DNA damage, contributing to cellular senescence and IHD progression^9,10^, but the degree to which these damaged nucleotides are removed by various DNA repair processes and whether they lead to enduring DNA changes that affect genome integrity or transcription remains uncertain. Recent studies have also highlighted the significant role of somatic mutations in hematopoietic cells, resulting in clonal hematopoiesis, to the development and progression of IHD^11,12,13^; however, to our knowledge, the impact of somatic mutations in cardiomyocyte and IHD has not been studied. To evaluate the hypothesis that particular mechanisms of genomic alterations influence IHD cardiomyocytes, we employed single-cell whole-genome sequencing (scWGS) on individual cardiomyocytes from the heart of individuals with IHD and heart-healthy controls to compare the burden, genomic pattern, and functional implication of somatic mutations linked to IHD. We also performed single-nucleus RNA sequencing (snRNA-seq) analysis from these hearts to evaluate the transcriptional changes between healthy and IHD cardiomyocytes.

## Results

### Somatic mutation accumulation in cardiomyocytes during aging

**Figure 1.**
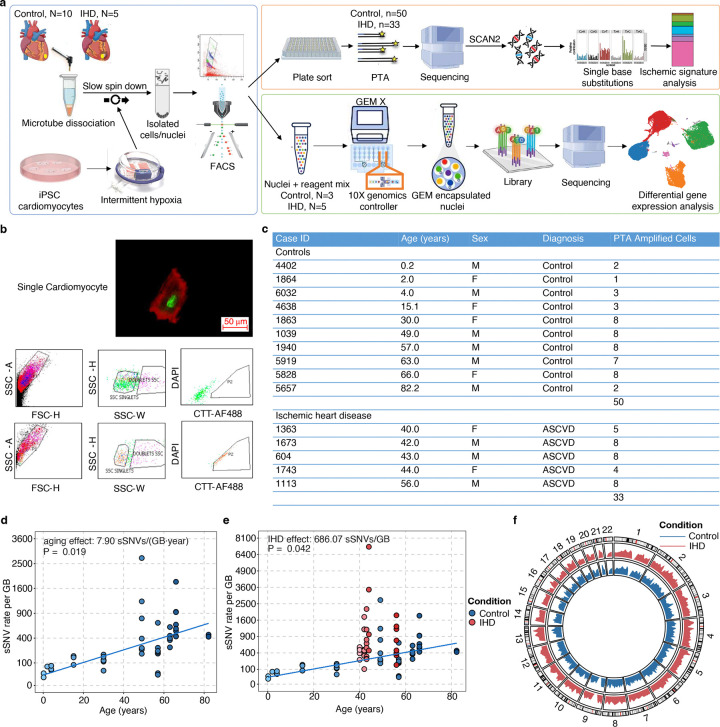
Genomic and transcriptomic profiling of single cardiomyocytes with and without IHD. **a**, Experimental outline for scWGS and snRNA-seq. Left ventricular cardiomyocytes and iPSC-derived cardiomyocytes were isolated using fluorescence-activated cell sorting (FACS), and their genomes were amplified using primary template amplification (PTA), followed by whole-genome sequencing and bioinformatics analyses for sSNV. The same cohort of heart samples was also subjected to snRNA-seq. **b**, FACS using AF488-conjugated anti-cTroponin (CTT) antibodies to label and separate cardiomyocytes from other cell types. **c**, Case information and number of cardiomyocytes analyzed in this study. **d-f**, Somatic mutational profiling of single cardiomyocytes with and without IHD. sSNV burden estimated in each single cardiomyocyte. Cardiomyocytes originating from the same individuals were shown with the same age. Control cardiomyocytes show an age-related accumulation of sSNVs (s = 7.90 sSNVs per GB per year, P = 0.019, linear mixed-effects model), shown as blue solid lines in **d** and **e**, while cardiomyocytes from IHD patients carried a significantly higher burden of sSNV than controls after correcting for age (IHD excess 686.07 sSNVs per GB, P = 0.042, linear mixed-effects model; **e**). **f**, Circos plot showing the genome-wide distribution of sSNVs in control and IHD cardiomyocytes. Blue arcs represent non-IHD controls, and red arcs represent IHD cases.

We performed an average of 30X scWGS (Supplementary Table 1) on cardiomyocytes isolated from the left ventricle of the heart in individuals with IHD and control individuals (Fig. 1a, Supplementary Table 2)^14,15^. We stained for the cardiomyocyte marker cardiac troponin T to mark cardiomyocytes and further gated only the diploid cardiac troponin T-positive cells (Fig. 1b). The genome of single cardiomyocyte was then amplified by primary template-directed amplification (PTA; Fig. 1a), which allows for highly uniform genome-wide coverage while mitigating known single-cell artifacts^16^. Somatic single-nucleotide variants (sSNVs) were called from each single cardiomyocyte using SCAN2^17^ (Supplementary Table 3), which utilizes local allele balance information inferred from germline mutations to distinguish real somatic mutations from amplification artifacts. In total, we profiled genome-wide sSNVs in 33 cardiomyocytes from five cases of IHD and 50 cardiomyocytes from ten non-heart-disease control individuals (Fig. 1c). Consistent with our previous findings^3^, our new PTA-based scWGS confirmed the age-dependent accumulation of somatic mutations in diploid cardiomyocytes. Across ten non-heart-diseased individuals, cardiomyocyte sSNVs increased significantly with age at a rate of 7.90 sSNVs per GB per year (P = 0.019, linear mixed-effects model), equivalent to ~46 sSNVs per diploid cardiomyocyte per year (Fig. 1d). Studies using clonally expanded cells from other human tissues have shown comparable yearly increases in sSNVs, ranging from 13 to 55 sSNVs per year, with higher rates in more rapidly dividing cell types^3,8,18–28^ (Supplementary Table 4).

### Increased burden of somatic mutation in ischemic heart cardiomyocytes

**Extended Data Figure 1.**
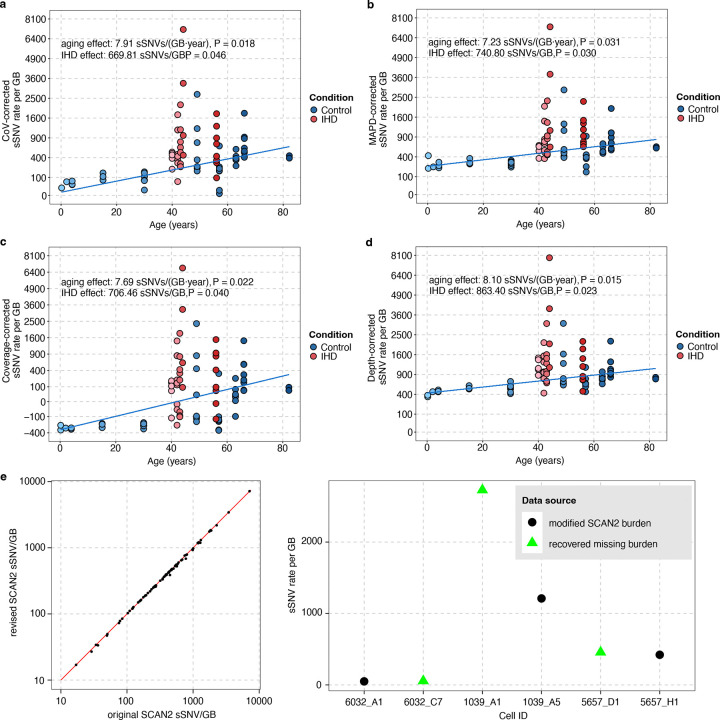
Evaluation of sSNV burden in control and IHD cardiomyocytes with the consideration of sequencing quality and amplification evenness metrics. **a-d**, Covariate-adjusted sSNV burden in each single cardiomyocyte. Cardiomyocytes originating from the same individuals were shown with the same age. sSNV burden robustly exhibited a clear age-related accumulation trend and significant increases in IHD cardiomyocytes compared to controls, after adjusting for CoV (**a**), MAPD (**b**), coverage (**c**), and depth (**d**) as covariate (linear mixed-effects model). **e**, Consistent sSNV burden estimates between the original and refined SCAN2 methods. **f**, Some cells that failed to have their sSNV burden estimated in the original SCAN2 method were recovered using the refined method, showing similar burdens as other cells originating from the same individuals.

We next assessed the burden of sSNVs in cardiomyocytes from the hearts of five individuals with IHD and found that IHD cardiomyocytes showed significantly more sSNVs than controls, after adjusting for age (P = 0.042, linear mixed-effects model), with an average excess of 686 sSNVs per GB (equivalent to ~4,010 sSNVs per cardiomyocyte; Fig. 1e). This excess varied across cardiomyocytes within the same donor and between donors, reflecting the variable disease pathology at both intra- and inter-individual levels in cardiomyocytes. The sSNV increase in IHD cardiomyocytes remained significant after normalizing for potential covariates including evenness of genomic amplification, sequencing depth, and coverage (Extended Data Fig. 1a–d). These results indicate that IHD cardiomyocytes contain thousands of additional sSNVs beyond what is expected for their age, suggesting that the ischemic disease condition exaggerates genomic DNA damage comparable to that which accumulates sSNVs normally over the course of more than a decade. The somatic mutations identified in IHD cardiomyocytes are pervasively distributed across the genome (Fig. 1f). Considering the huge increase of sSNV burden in IHD cardiomyocytes, the broad genomic distribution of variants suggests that, rather than constituting a specific initial event in a particular chromosome or gene loci for IHD pathogenesis, somatic mutations are more likely to be resulting from other events that initiate IHD and insinuate the mutagenic processes. We also observed no significant effect of an individual’s sex on the accumulation of sSNVs (P = 0.183, linear mixed-effects model).

### Distinct patterns of somatic mutations in ischemic heart cardiomyocytes

**Figure 2.**
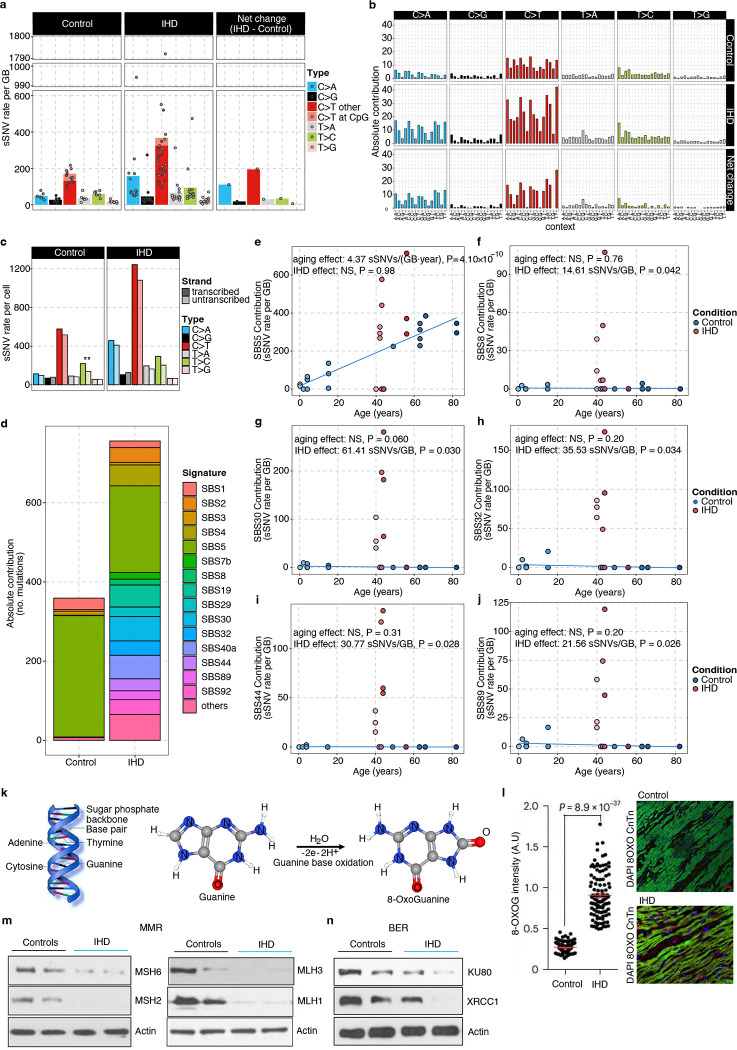
Somatic mutational patterns and signatures in control and IHD cardiomyocytes. **a-b**, Base Substitution distribution of the sSNVs in individuals with IHD versus age-matched control. C>A and non-CpG C>T substitutions are predominant. **c,** Transcriptional strand bias for sSNVs in transcribed regions. Significant strand bias is observed only for T>C mutations in control cardiomyocytes (P = 0.009, two-sided Wilcoxon test). **d**, The absolute contribution of each COSMIC signature to the mutational spectra of control and IHD cardiomyocytes, shown as stacked bar plots. **e-j**, Signature-specific sSNV burden identified using SigNet. The clock-like signature SBS5 demonstrated a clear age-related accumulation trend but showed no significant increase in IHD (**e**), whereas several other signatures including SBS8 (**f**), SBS30 (**g**), SBS32 (**h**), SBS44 (**i**), and SBS89 (**j**) showed significant increase in IHD but no effect on age. **k**, Schematic representation of 8-oxoG generation through oxidative DNA damage. **l**, Oxidative damage in IHD cardiomyocytes, using 8-oxoG immunofluorescence. Data points represent mean absorbance units (AU) ± s.e.m. of n = 100 cardiomyocytes per case. Trend lines show linear mixed-effects regression (IHD versus control: P = 8.9 × 10^−37^). Inset shows representative immunofluorescence images; cardiomyocytes (CnTn; green) and oxidized guanine (8-oxoG; Red). Scale bars, 100μm. **m-n**, Immunoblot analysis for MMR and BER markers indicating decreased expression of MHS6/2, MLH1/3, XRCC1, and Ku80 in IHD hearts compared to control.

Mutational signatures, the characteristic patterns of base substitution in the trinucleotide genomic context, have emerged as powerful instruments for elucidating the somatic mutational mechanisms that contribute to aging and the pathogenesis of various diseases^29^. Using 90,249 sSNVs identified from 33 cardiomyocytes, we studied the relative contribution of different substitution types in control and IHD cardiomyocytes. We observed that C>T and C>A mutations were the primary contributors to the excess SNVs in IHD cardiomyocytes (Fig. 2a–b). We also checked the strand bias of sSNVs between control and IHD cardiomyocytes but observed no significant differences, except for a modest strand bias in T>C sSNVs for control cardiomyocytes (Fig. 2c).

Next, we conducted a mutational signature analysis to identify whether specific mutational mechanisms lead to somatic alterations in the genomes of IHD cardiomyocytes. We evaluated the contribution of COSMIC single base substitution (SBS) signatures to the mutational spectra of control and IHD cardiomyocytes, and identified multiple active COSMIC signatures including SBS5, 8, 30, 32, 44, and 89 (Fig. 2d–j). Among them, the abundance of SBS5-related sSNVs increased with age across all IH and control cardiomyocytes (Fig. 2e); SBS5 is a clock-like signature observed universally in all types of cancer and normal cells^30^, reflecting an intrinsic hallmark of genomic aging in cardiomyocytes.

On the other hand, SBS8, 30, 32, 44, and 89 were significantly more abundant in IHD cardiomyocytes compared to controls (Fig. 2f–j) and collectively accounted for 34% of the excess sSNVs observed in IHD cardiomyocytes (Fig. 1e), underscoring disease-specific mutational mechanisms. SBS8, associated with defects in the DNA repair of oxidized guanine, has been reported at low but highly variable levels in normal cardiomyocytes, with some age-related accumulation^3^. Elevated DNA oxidation, previously reported in IHD in both human and animal models^31^, may drive the excess of SBS8 in IHD. To further investigate this, we examined nucleotide oxidative damage in individual cardiomyocytes. In response to ROS activation the hydroxyl radical directly attacks the double bond in guanine to become 8-oxoG (Fig. 2k). Using 8-oxoguanine (8-oxoG), a biomarker of oxidative stress and DNA damage, immunofluorescence microscopy revealed significantly higher 8-oxoG levels in IHD cardiomyocytes compared to controls (P = 8.9 × 10^−37^; Fig. 2l). This suggests increased oxidative damage contribute to C>A mutations and SBS8 in IHD cardiomyocytes.

SBS30 and SBS44 were specifically elevated in IHD cardiomyocytes (Fig. 2g, i); both of which have been linked to DNA repair deficiencies^32^. SBS30 is associated with impaired base excision repair (BER) due to *NTHL1* dysfunction and unexcised uracil residues from uracil-DNA glycosylase (UNG) deletion^30^, via the BER pathway. SBS44, meanwhile, is associated with the defective mismatch repair (MMR) pathway and resembles signatures produced by knocking out several DNA repair genes such as *MSH2*, *MLH1*, and *MSH6*^33^. Thus, we investigated the expression of MMR and BER pathway genes in our cohort using immunoblotting. We observed a consistent and significant decrease in the expression of MMR genes in ischemic heart (Fig. 2m), while the downregulation of BER genes was donor-specific (Fig. 2n). Our results suggest that the mutational burden associated with SBS30 and SBS44 likely results from oxidative stress-induced mutagenesis, where DNA repair mechanisms are ineffective in IHD cardiomyocytes.

IHD cardiomyocytes also exhibited an increased burden of SBS32-related sSNVs (Fig. 2h), which have been associated with the pretreatment of immunosuppressants^32^. We hypothesize that excessive systemic inflammation due to ischemia may create a similar environment in heart tissue, as hyper-inflammation and immune suppression are considered subsequent phases in the compensatory anti-inflammatory response^34^; this might explain the accumulation of immunosuppressant-related SBS32 in IHD cardiomyocytes.

**Extended Data Figure 2.**
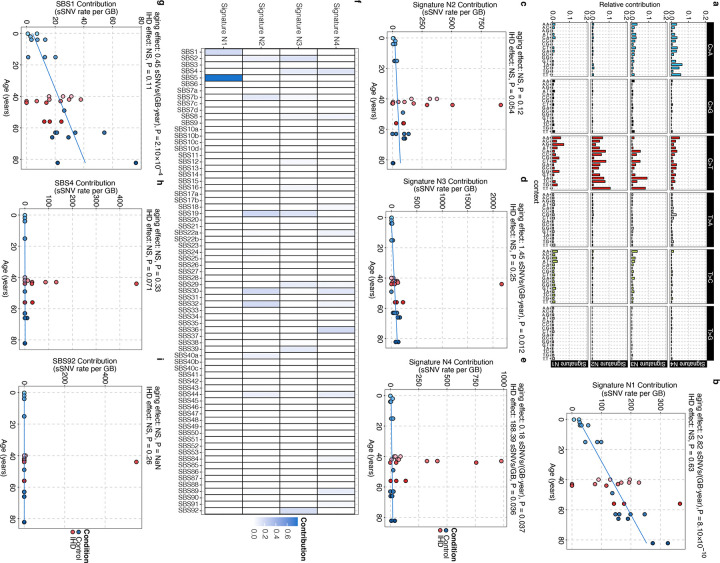
*De novo* mutational signature analysis for cardiomyocyte sSNVs. **a**, Four *de novo* signatures (N1, N2, N3, and N4) were identified from the aggregated mutational spectra across all control and IHD cardiomyocytes using non-negative matrix factorization (NMF, implemented in MutationalPatterns). **b-e**, *de novo* signature-specific sSNV burden estimated using MutationalPatterns. Signatures N1, N3, and N4 exhibited a clear age-related accumulation trend, with only signature N4 showing a significant increase in IHD (linear mixed-effects model). **f**, Decompose N1, N2, N3, and N4 into COSMIC signatures using SigNet. **g-i**, Signature-specific sSNV burden was analyzed using SigNet. Signature SBS1 demonstrated a clear age-related accumulation trend but showed no significant increase in IHD. Tobacco smoking associated signatures SBS4 and SBS92 showed neither a significant age-related accumulation trend nor a significant increase in IHD.

Our *de novo* signature decomposition identified four distinct mutational signatures (Extended Data Fig. 2a), with N1 accumulating with aging and N2-N4 showing increased prevalence in IHD (Extended Data Fig. 2b–e). These *de novo* signatures were attributed to similar SBS identified by COSMIC decomposition, demonstrating consistency (Extended Data Fig. 2f). Given that increased ROS and oxidative nucleic acid lesions have been reported in IHD^35^, a plausible mechanism for the accumulation of mutations associated with SBS30, 44, and 8 in IHD is that increased oxidative damage creates a cytotoxic environment that overwhelms NER, BER, and MMR which could also be attenuated in IHD.

### Potential consequences of somatic mutations in ischemic heart genome

**Figure 3.**
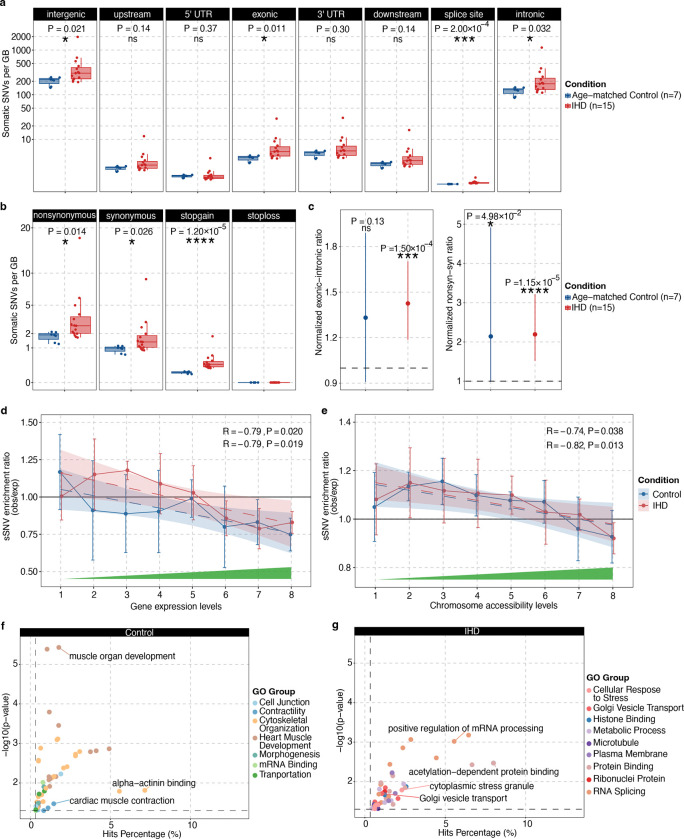
Distribution of cardiomyocyte sSNVs with gene-level annotation. **a-b**, Distribution of sSNVs across genic regions (**a**) and across functional categories (**b**) in IHD and age-matched control. IHD cardiomyocytes harbor a significantly higher burden of sSNVs predicted to be protein-altering (P < 0.05, two-sided Wilcoxon test). **c**, Natural selection against sSNVs assessed by the exonic-to-intronic ratio and nonsynonymous-to-synonymous ratio (dN/dS), normalized by germline sSNVs. Both control and IHD cardiomyocytes exhibit ratios greater than one, with stronger significance in IHD cardiomyocytes (P = 1.50 × 10^−4^ and 1.15 × 10^−5^, two-sided Fisher’s exact test), suggesting relaxed negative selection on somatic mutations compared to germline mutations. **d-e**, Association between somatic mutations and gene transcription or chromosome accessibility. Solid lines represent the sSNV enrichment ratio with error bars representing the standard deviation, while dashed lines indicate the least-squares regression fit across eight groups classified by their transcription or chromosomal accessibility levels. In both control and IHD cardiomyocytes, sSNVs tend to be enriched in regions with low gene expression and relatively closed chromatin. **f-g**, GO enrichment analysis of genes harboring deleterious sSNVs in control cardiomyocytes (**f**) or IHD cardiomyocytes (**g**). Control cardiomyocytes show an enrichment of deleterious somatic mutations in pathways related to heart muscle development, cytoskeletal organization, and cardiac muscle contraction. In contrast, IHD cardiomyocytes exhibit an enrichment of somatic mutations in pathways associated with cellular response to stress, regulation of mRNA splicing, and acetylation-dependent protein binding.

Next, we aim to study the potential functional impacts of somatic mutations in cardiomyocytes. We observed that IHD cardiomyocytes harbored significantly more sSNVs than age-matched controls in exonic and splice-site regions (P = 0.011 and 2.00 × 10^−4^, two-tailed Wilcoxon test; Fig. 3a) and for nonsynonymous and stopgain mutations (P = 0.014 and 1.20 × 10^−5^; Fig. 3b), suggesting that the excess sSNVs in IHD cardiomyocytes are more likely to impact gene functions. Additionally, we found that somatic mutations in cardiomyocytes exhibited higher exonic-to-intronic and nonsynonymous-to-synonymous ratios compared to germline mutations, and these ratios tend to be further elevated in IHD cardiomyocytes (Fig. 3c), indicating relaxed negative selection on somatic mutations, particularly in IHD cardiomyocytes.

Further, we investigated the relationship between cardiomyocyte somatic mutations and gene expression levels using cardiomyocyte snRNA-seq data for eight subjects in our cohort. After accounting for local mutation detection sensitivity in scWGS (see Methods for details), we observed a negative association between sSNV density and transcription levels in both IHD (R = −0.79, P = 0.019) and control cardiomyocytes (R = −0.079, P = 0.020; Fig. 3d). Examination of cardiomyocyte-specific snATAC-seq data further confirmed our findings, where somatic mutations were enriched in lower chromatin accessibility regions in both IHD (R = −0.82, P = 0.013) and control cardiomyocytes (R = −0.74, P = 0.038; Fig. 3e). These findings suggest that somatic mutations in cardiomyocytes preferentially occur in untranscribed and less accessible genomic regions, which is in contrary with observations in another non-proliferative cell type, neurons^28^.

**Extended Data Figure 3.**
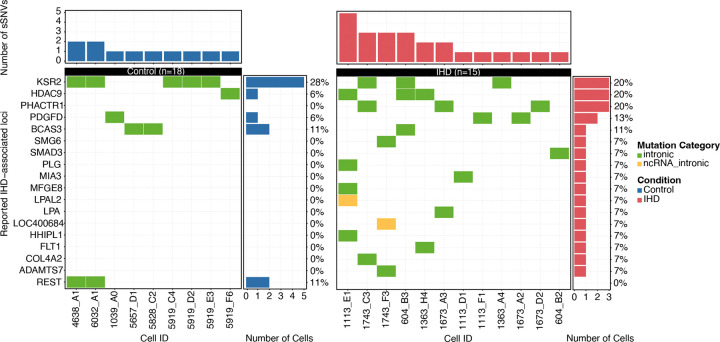
Cardiomyocyte sSNVs in previously reported IHD-associated loci. A limited number of overlaps were identified, with all overlapping sSNVs located exclusively in intronic regions.

Somatic mutations that alter amino acids can lead to cardiomyocyte dysfunction or loss through several processes, including direct transcriptional disruption and changes in protein function. We first examined whether any of these sSNVs were located within previously reported IHD-associated loci^36,37^. We identified very few overlaps, all of which were confined to intronic regions (Extended Data Fig. 3), suggesting that somatic mutations do not frequently affect these conventional IHD risk genes. To systematically evaluate the impact of somatic mutations on cardiomyocyte function and to explore the potentially novel biology of IHD, we performed Gene Ontology (GO) enrichment analysis on predicted deleterious sSNVs in control and IHD cardiomyocytes (Supplementary Table 5). Control cardiomyocytes showed enrichment in distinct pathways, such as heart muscle development and cardiac muscle contraction (Fig. 3f); in contrast to IHD cardiomyocytes where we found enrichment of somatic mutations in pathways involved in cellular stress responses, regulation of mRNA splicing, and acetylation-dependent protein binding (Fig. 3g). Notably, while many pathways were novel to IHD, some, like the integrated stress response (ISR) and RNA regulation, have been previously implicated. We observed deleterious mutations in key ISR genes—*CIRBP*^38^, *NCL*^39^, and *FXR1*^40^—that regulate mRNA stability, processing, and translation under stress^41^ in IHD cardiomyocytes. *CIRBP* stabilizes and modulates genes involved in cell survival and death^38^, whereas *NCL* and *FXR*1 regulate ribosome biogenesis, chromatin structure, and stress granule formation^42,43^. Another enriched pathway involves vesicular trafficking and intracellular transport. In IHD cardiomyocytes, deleterious mutations have been identified in several key genes: *IGF2R,* which facilitates lysosomal enzyme trafficking^44^, *DENND5A,* which regulates Rab GTPases for vesicle movement^45^, and *ATP2C1*, which modulates calcium-dependent vesicle formation^46^. The cumulative impact of somatic mutations in these genes may impair cellular adaptation to ischemic stress, protein quality control, and calcium signaling, contributing to greater susceptibility to ischemic damage and impaired cardiac repair in IHD cardiomyocytes. The interplay of these genes, particularly in ISR pathways, highlights their critical roles in vesicular trafficking, calcium homeostasis, and stress adaptation, which are essential for the heart’s response to ischemic insults.

### Somatic mutations in iPS-derived hypoxic cardiomyocytes

**Figure 4.**
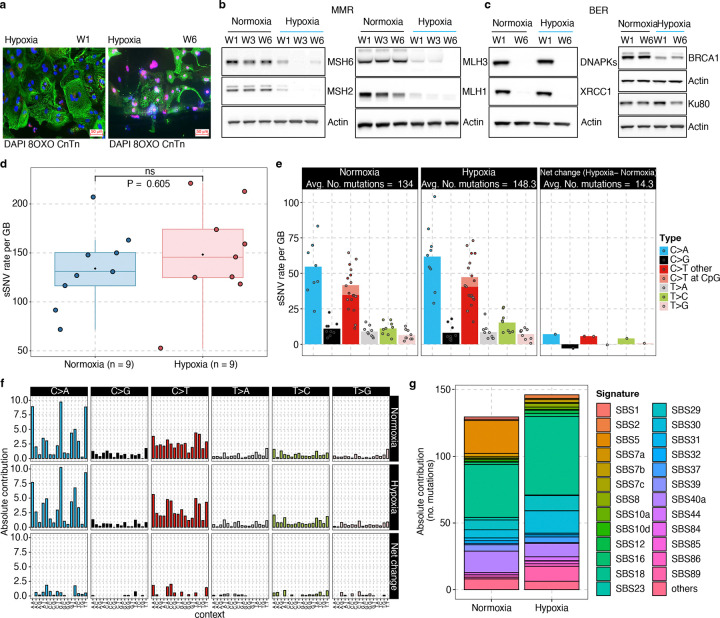
Somatic mutational analysis of iPS-derived hypoxic cardiomyocyte. **a**, Representative immunofluorescence images for hiPSC-CMs (cTnT; green) and oxidized guanine (8-oxoG; red). After 14 days of differentiation, hiPSC-CMs were subject to intermittent hypoxia (48 hrs. hypoxia followed by 96 hrs. normoxia) for six weeks. Oxidative damage in hypoxic iPSC-CM, using 8-oxoG immunofluorescence on Week 1 (W1) through Week 6 (W6) of hypoxia. Scale bars, 100μm. **b-c**, Immunoblot analysis for MMR and BER markers in normoxia and hypoxia iPSC-CM over six weeks indicating decreased expression of MHS6/2, MLH1/3, XRCC1, to no change in Ku80 in hypoxic hiPSC-CM. **d**, sSNVs burden comparison between hiPSC-CMs after 6 weeks of normoxia or hypoxia. Each data point represents a single hiPSC-CM. We observed a trend toward a higher sSNV burden under hypoxic conditions, although the difference did not reach statistical significance (P = 0.605, two-sided Wilcoxon test). **e-f**, Base substitution distribution of the sSNVs in hypoxic iPSC CMs versus normoxic iPSC CMs. C>A and non-CpG C>T are predominant. **g**, The absolute contribution of each COSMIC signature to the mutational spectra of hypoxic and normoxic cardiomyocytes, shown as stacked bar plots.

To investigate whether hypoxic conditions—a hallmark of IHD—contribute to the increased sSNV burden and associated substitution patterns observed in IHD cardiomyocytes, we developed an *in-vitro* ischemic model using iPSC-derived cardiomyocytes (hiPSC-CMs) cultured in 2D and characterized it following intermittent hypoxia treatment (Fig. 1a; see Methods for details). After six weeks of intermittent hypoxia, we measured the levels of 8-oxoG in hiPSC-CMs as guanine has the lowest redox potential making 8-oxoG the most common oxidized form. An increased 8-oxoG, indicating oxidative stress and DNA damage comparable to that in human IHD cardiomyocyte findings (Fig. 4a). Protein expression analysis showed a downregulation of key components in the MMR (*MSH2*, *MSH6*, *MLH1*, *MLH3;* Fig. 4b) and BER (*DNPKcs*, *XRCC1*, *BRCA;* Fig. 4c) pathways, while *Ku80* expression remained unchanged (Fig. 4c). We hypothesize that hypoxia-induced metabolic reprogramming leads to increased production of ROS, which overwhelms the BER pathway and potentially impairs its function, potentially by preventing the accumulation of XRCC1 at DNA single-strand breaks^47,48^. In contrast, Ku80 has been reported to localize to the cell surface under hypoxic conditions, enhancing cell adhesion and potentially promoting metastasis^49^. This differential regulation of Ku80, compared to other DNA repair genes, highlights the complex and selective nature of cellular adaptation to hypoxic stress, where maintaining some level of DNA repair capability may be crucial for cell survival in a low-oxygen environment. However, it might also reflect cellular immaturity of hiPSC-CMs, which have a high regenerative potential when compared to adult human CMs^50^.

**Extended Data Figure 4.**
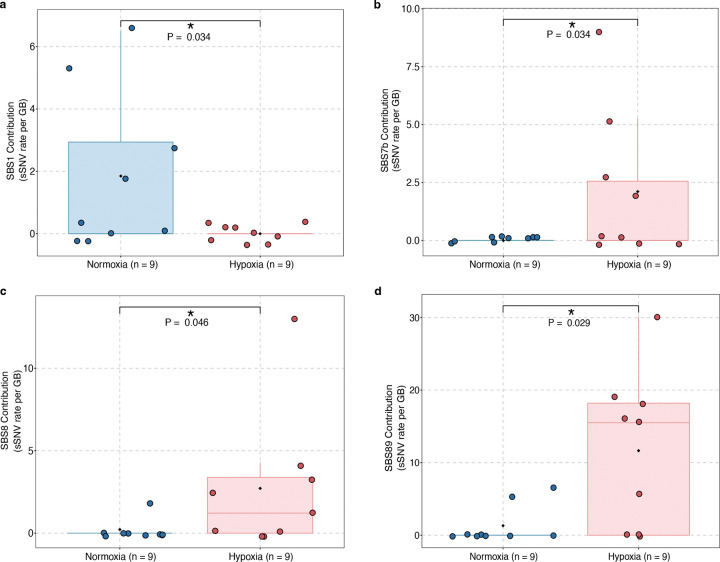
Signature-specific sSNV burdens in hypoxic and normoxic hiPSC-CMs. **a**, SBS1 contribution is significantly higher in normoxic hiPSC-CM (P = 0.034, two-sided Wilcoxon test). **b-d**, SBS7b, SBS8, and SBS89 contribution are significantly higher in hypoxic hiPSC-CM (P = 0.034, P = 0.046, and P = 0.029, respectively, two-sided Wilcoxon test).

A comparison of the sSNV burden and substitution types between normoxic and hypoxic hiPSC-CMs revealed an insignificant increase in sSNV burden in hiPSC-CMs (Fig. 4d and Supplementary Table 6) and a predominant accumulation of C>T and C>A mutations (Fig. 4e–f), consistent with the patterns we observed in IHD cardiomyocytes. Notably, hiPSC-CMs exhibited a higher proportion of C>A mutations compared to IHD cardiomyocytes, likely reflecting cell culture-associated substitution patterns previously reported in other cell types^51,52^. Using SigNetProfilerExtractor^53^ to quantify contributions of COSMIC mutational signatures, we identified multiple active signatures in either normoxic or hypoxic hiPSC-CM, including SBS1, 5, 8, 7b, 18, 30, and 89 (Fig. 4g, Extended Data Fig. 4a–d). Most of these signatures were also observed in IHD cardiomyocytes. Our analysis was constrained by the relatively low number of sSNV identified in hiPSC-CMs supporting our hypothesis that DNA damage is mainly induced by oxidative stress in human CMs. Even though they are limited by cell culture conditions and immaturity of hiPSC-CMs, these cells still display regenerative potential and therefore some DNA repair activity as seen by sustained expression of the KU80 protein. However, a consistent trend indicating an increased mutational burden with analogous mutational spectra substantiates our hypothesis that oxidative stress-induced mutagenesis, coupled with inadequate DNA repair in hypoxic cardiomyocytes, results in the excess of somatic mutations in IHD.

### Transcriptional changes in ischemic heart cardiomyocytes

**Extended Data Figure 5.**
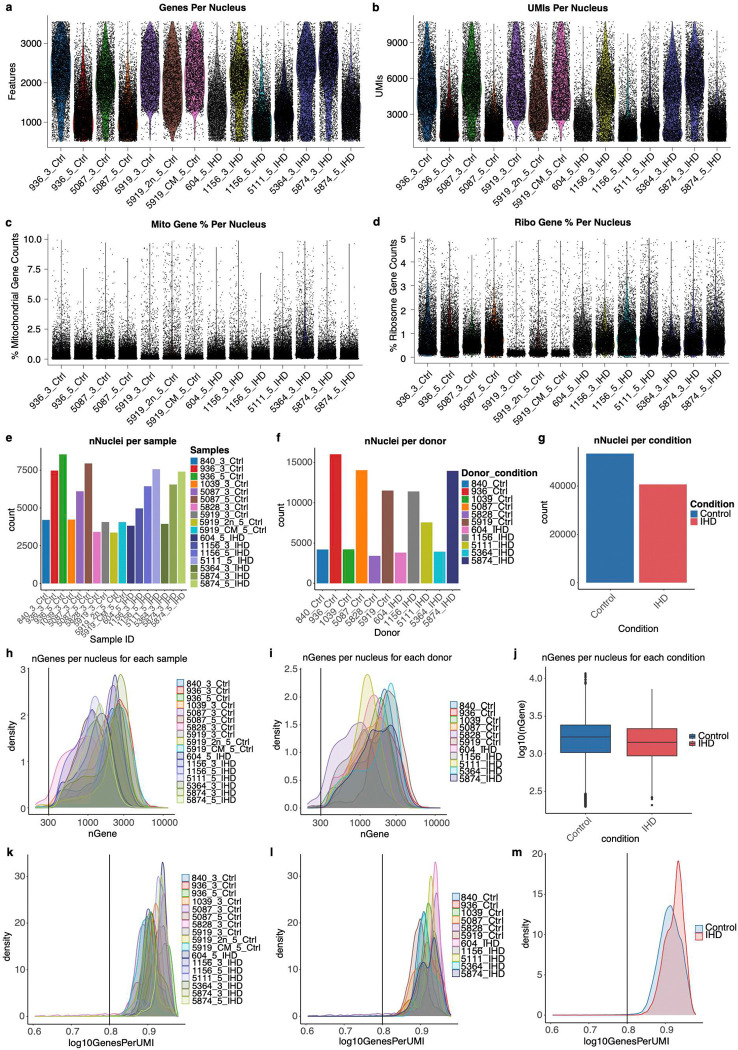
snRNA-seq quality control metrics. **a-d**, Violin plots depicting the number of detected genes per nucleus (**a**), UMI per nucleus (**b**), mitochondrial gene percentage per nucleus (**c**), and ribosomal gene percentage per nucleus (**d**). **e-g**, Number of nuclei in snRNA-seq of this study, grouped by samples (**e**), donors (**f**), or conditions (**g**). **h-j**, Number of genes per nucleus in snRNA-seq of this study, grouped by samples (**h**), donors (**i**), or conditions (**j**). **k-m**, Number of genes per UMI in snRNA-seq of this study, grouped by samples (**k**), donors (**l**), or conditions (**m**).

**Figure 5.**
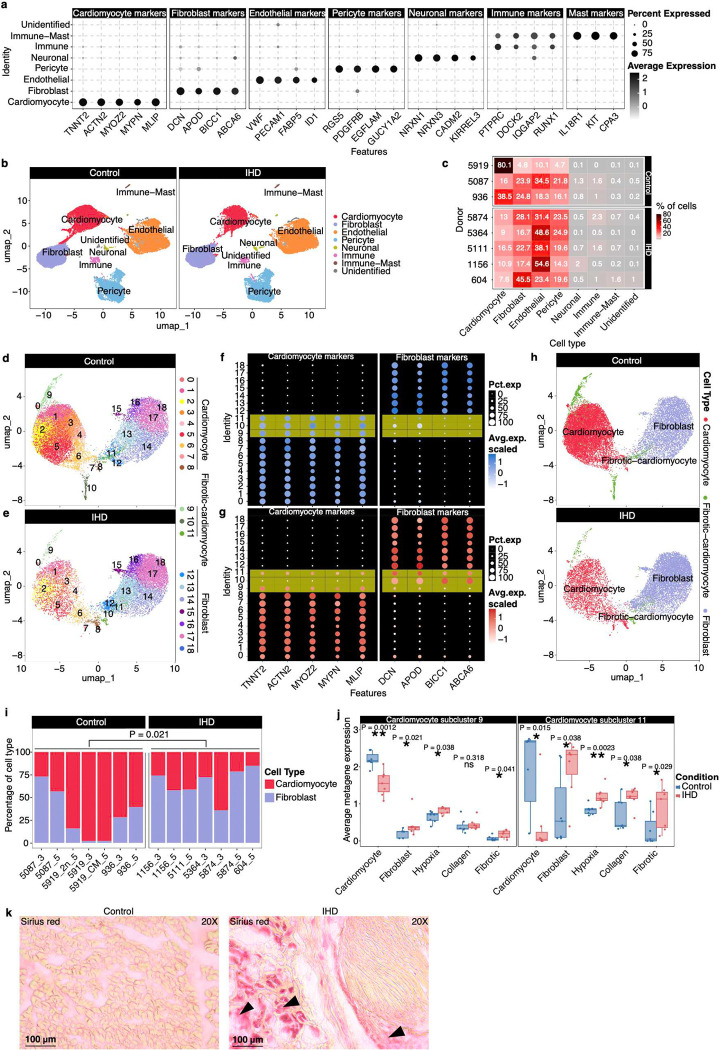
Phenotypic shifts and the emergence of disease-associated fibroblasts in IHD. **a**, Expression of characteristic marker genes in each identified cell population. **b**, UMAP clustering based on marker gene expression for control and IHD heart tissues after QC, data filtering, and Harmony integration. **c**, Relative proportion of different cardiac cell types for control and IHD samples. **d-e**, Unsupervised sub-clustering of cardiomyocytes and fibroblasts identifies 19 unique clusters within the integrated dataset across control (**d**) Control and IHD cardiomyocytes (**e**). **f-g,** Expression of cardiomyocyte and fibroblast marker genes across these 19 clusters in control (**f**) and IHD (**g**) samples. Three clusters (9, 10, and 11) that predominantly express cardiomyocyte markers in the control heart shift to expressing fibroblast markers in the IHD heart (fibrotic-cardiomyocyte). **h**, Identified cardiomyocyte, fibrotic-cardiomyocyte, and fibroblast populations in control and IHD heart. **i**, Relative proportion between cardiomyocytes and fibroblasts in control and ischemic heart samples. IHD individuals showed decreased proportions of cardiomyocytes and increased proportions of fibroblasts (P = 0.021, two-tailed Wilcoxon test). **j**, Average metagene (as listed in Supplementary Table 9) expression in clusters 9 and 11 from control and IHD samples, indicating downregulation of cardiomyocyte genes, upregulation of fibroblast, hypoxia, and collagen genes. Statistical significance was evaluated using a two-sided Wilcoxon test. **k,** Immunohistochemistry using Sirius red staining; collagen fibers in red and muscle fibers in yellow.

To characterize the cellular and transcriptional landscapes of healthy and ischemic human hearts and investigate the impact of hypoxia on these landscapes, we obtained left ventricular cardiac tissue specimens from three controls and five individuals with IHD. These samples were processed for snRNA-seq using the 10x Genomics Single Cell platform (Fig. 1a). After quality control, the final integrated dataset comprised 66,630 nuclei, representing seven major cell types: cardiomyocytes, fibroblasts, endothelial cells, pericytes, neuronal cells, immune cells, and mast cells, where cell identities were confirmed using cell-specific marker genes and transcriptional signatures (Fig. 5a–c, Extended Data Fig. 5a–m). From the UMAP (Fig. 5b), the cardiomyocyte and fibroblast clusters showed differences in the clustering pattern in IHD compared to controls, and the close proximity of the clusters, with a certain overlap, intrigued us to focus on this population which is also in line with previous findings where ischemic human hearts become more fibrotic following injury^54^. Thus, we performed our subsequent analysis on specific transcriptional changes between cardiomyocytes and fibroblasts. This analysis was performed separately in control and IHD cohorts to account for the possibility that the relationship between cell composition and gene expression may differ in health and heart failure.

Unsupervised clustering identified twelve cardiomyocyte and seven fibroblast subclusters across control (Fig. 5d) and IHD (Fig. 5f) samples. In control hearts, cardiomyocyte and fibroblast subclusters were clearly distinct, characterized by the nearly exclusive expression of their respective marker genes (Fig. 5e). However, in IHD, three cardiomyocyte subclusters (Subclusters 9–11) exhibited reduced expression of cardiomyocyte markers alongside increased expression of fibroblast markers (Fig. 5g), indicative of a fibrotic cardiomyocyte transition (Fig. 5h). This transition was further supported by our observation where IHD individuals showed decreased proportions of cardiomyocytes and increased proportions of fibroblasts (P = 0.021, two-tailed Wilcoxon test; Fig. 5i). Additionally, Subclusters 9 and 11 showed elevated expression of genes associated with hypoxia, collagen production, and fibrosis (Fig. 5j), which could be explained by higher expression of *COL4A2, PDGFB,* and MMP2. Increased deposition of collagen, observed with Sirius Red staining, confirmed the fibrotic shifts in IHD (Fig. 5k, Supplementary Table 7).

**Figure 6.**
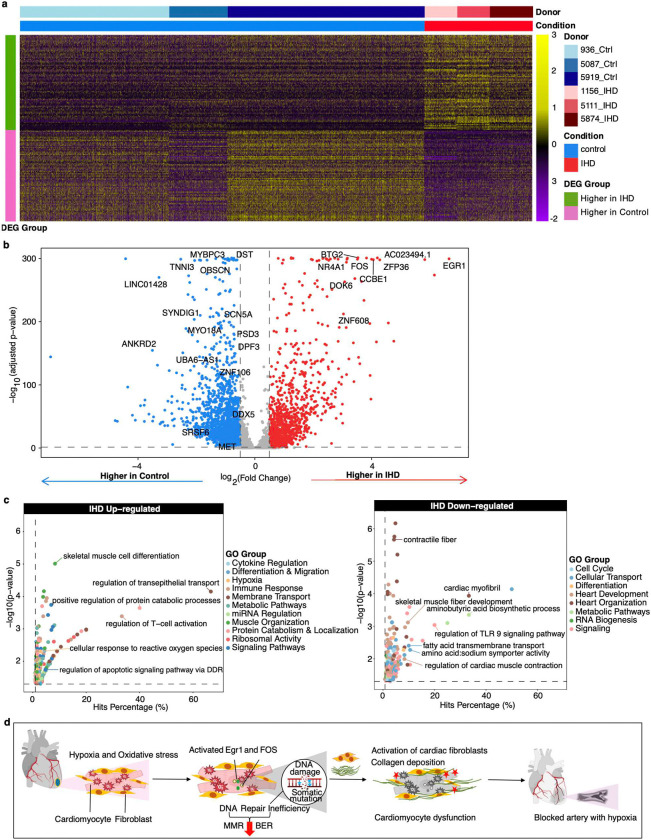
Differential gene expression between control and IHD cardiomyocytes indicates ISR activation in response to hypoxic stress, leading to downregulation of DNA damage repair pathway genes. **a**, Heatmap of 164 differentially expressed genes (DEGs) in control and IHD cardiomyocytes. The scaled expression levels for each gene and cardiomyocyte are displayed, grouped by samples and clinical conditions. **b**, Volcano plot of these DEGs between ischemic and control heart cardiomyocytes. The complete list of upregulated and downregulated DEGs is provided in Supplementary Table 7. **c**, Pathway enrichment analysis for DEGs. Genes related to hypoxia, inflammation, and signal transduction are upregulated in IHD cardiomyocytes, while genes involved in maintaining contractile fiber, cardiomyocyte contraction, and skeletal muscle fiber development are downregulated. **d**, A model illustrating the role of somatic mutations in IHD development. Oxidative stress and hypoxia trigger events leading to ROS buildup. This activates *EGR1* and *FOS*, downregulates DNA repair genes, and increases DNA damage in ischemic cardiomyocytes. MMR and BER dysregulation might cause base misincorporation during repair, contributing to the transition from DNA damage to mutation. These events result in heightened cellular susceptibility through processes such as activating ISR-related genes, promoting collagen buildup, and fibroblast proliferation, which contribute to cardiac dysfunction and occluded arteries, hallmark features of IHD.

Further differential gene expression analysis between control and IHD cardiomyocytes identified 70 up-regulated and 50 down-regulated genes in IHD (Fig. 6a, b, Supplementary Table 8). Pathway analysis of these genes reveals a complex network of cellular processes affected by ischemic conditions, with critical implications for cardiac function and disease progression (Fig. 6c, Supplementary Table 9). Notably, genes involved in immediate-early responses (e.g., *EGR1*, *FOS*, *JUNB*, *NR4A1*) and stress response (e.g., *CRYAB*, *BTG2*, *GADD45G*, *HSPB3*) exhibited increased expression in IHD, reflecting cellular adaptations to ischemic stress. These adaptations may lead to up-regulation of genes involved in signal transduction, cell communication (e.g., *DOK6*, *LGR4*, *PKNOX2*, *RORA*), apoptosis, cell survival (e.g., *PTEN*, *PDGFD*, *MAP1LC3A*), and structural proteins (e.g., *MYL6*, *ACTB*, *ACTG1*), collectively impacting cardiac function, energy metabolism, and cellular integrity. Additionally, the inflammatory milieu, characterized by elevated production of cytokines and altered expression of *CD81* and *CD46*, aligns with previous findings that associate these markers with the pathogenesis of atherosclerosis and IHD^55,56^. Our analysis also indicates down-regulation of genes involved in cell cycle (e.g., *BACH1*, *BCL2*), cytoskeletal organization (e.g., *TMEM38B*, *SCN5A*), and DNA repair pathway (e.g., *SRSF6*^57,58^, *DDX5*^59^, and *MET*^60^), which is consistent with our mutational signature analysis showing increased oxidative stress and defective DNA repair mechanisms in IHD cardiomyocyte. These findings highlight the complex interplay between hypoxia and oxidative stress-mediated changes in the genome and transcriptome. Increased sSNV burden, DNA damage, and transcriptional stress activation contribute to a more fibrotic heart, with loss of cardiomyocytes and progression to IHD (Fig. 6d). These insights offer potential targets for therapeutic interventions aimed at enhancing DNA repair capacity and mitigating the effects of genomic instability in cardiac tissues.

## Discussion

Our study reveals that cardiomyocytes from individuals with IHD accumulate DNA alterations, resulting in an exaggerated burden of permanent somatic mutations compared to age-matched control hearts. While normal cardiomyocytes accumulate mutations at a rate of approximately 46 sSNVs per year, reflecting the age-related “clock-like” signature (SBS5)^32^, IHD cardiomyocytes display an additional 4,010 sSNVs indicative of more extensive genomic damage. The genomic pattern of accumulated sSNVs in IHD cardiomyocytes appears to be distinct from normal aging, as suggested by the abundance of signatures SBS8, 30, 32, and 44, which are present but limited in the control heart cardiomyocytes. This specific pattern in IHD cardiomyocytes also offers us novel insights into the genomic landscape of IHD condition and its potential implications for cardiac function and disease progression. Our findings reveal a notable increase of C>A substitutions, which are associated with SBS8^32^, a signature linked to oxidative damage and defects in repairing oxidized guanine. The increased sSNV burden in SBS30 and SBS44 is associated with deficiencies in DNA repair pathways, particularly BER and MMR^32^. An increase in SBS32 is potentially related to systemic inflammation and compensatory immune responses. These findings suggest that the increased mutational burden in IHD cardiomyocytes is driven by a combination of oxidative stress and impaired DNA repair mechanisms, creating a cytotoxic and inflammatory environment in the myocardium. The elevated levels of oxidative DNA damage, as evidenced by increased 8-oxoG, coupled with the downregulation of DNA repair genes in IHD cardiomyocytes, paint a picture of cellular stress overwhelming the normal repair capacity. This scenario is consistent with the chronic ischemic conditions experienced in IHD, where hypoxia-induced oxidative stress likely plays a central role in generating DNA damage^61,62^.

Gene ontology analysis on these sSNVs revealed that mutations in IHD cardiomyocytes affect pathways related to stress response, RNA regulation, vesicular trafficking, and calcium signaling. These alterations could impair cardiac repair mechanisms and contractility, providing a molecular basis for the progressive nature of IHD and the challenges in restoring normal cardiac function. Subsequent snRNA-seq analysis of IHD and control hearts corroborates these results by demonstrating the activation of immediate-early and stress response pathway genes, along with the downregulation of structural proteins and DNA damage repair pathway genes, collectively impacting the cellular integrity and, ultimately, cardiac function. Our study further suggests that, while the hiPSC-CM ischemia model exhibits many similarities to the human heart in terms of sSNV patterns, it may not fully recapitulate the sSNV burden or the chronic ischemic conditions observed *in vivo*, potentially due to the relatively short culture duration and artifacts introduced by the cell culture environment.

Our findings provide a deeper understanding of the molecular mechanisms underlying IHD progression, highlighting the role of somatic mutations in cardiomyocyte dysfunction. Key pathways disrupted by somatic mutations, such as DNA repair mechanisms and oxidative stress response, emerge as potential therapeutic targets. Interventions aimed at enhancing DNA repair capacity or mitigating oxidative damage could slow disease progression and improve cardiac function. The observed variability in mutational burden and signatures among IHD patients suggests that personalized therapies tailored to individual genomic profiles may offer advantages over generalized treatment approaches.

Further studies explicitly comparing sSNV across different heart cell types, including fibroblasts, endothelial cells, and immune cells, could provide a more comprehensive view of the mosaic cellular dynamics in IHD and may elucidate the impacts of these interconnected phenomena on disease progression. Other types of somatic mutations—such as small insertions and deletions, structural variations, and retro transposition events merit further exploration as advancements in technology enable more detailed analyses.

## Methods

### Sample preparation

This study was conducted with approval from the Boston Children’s Hospital institutional review board and Boston Children’s Hospital institutional biosafety committee, following standardized protocols and guidelines from the National Institutes of Health NeuroBioBank. The research was conducted at the Boston Children’s Hospital with approval from the Committee on Clinical Investigation, using deidentified human tissue and data obtained from the NIH NeuroBioBank at the University of Maryland. The quality of the DNA in the tissue samples was evaluated by isolating the DNA and conducting gel electrophoresis and a Genomic DNA Screen Tape Station. Only samples with intact DNA were selected for further study.

### Cardiomyocyte isolation from human myocardium

Cardiomyocytes were isolated using a protocol described previously^15^. 100 mg of tissue from the left ventricle was finely chopped and mixed with a cold solution of lysis buffer (5 ml of ice-cold lysis buffer (0.32 M sucrose, 5 mM CaCl2, 3 mM magnesium acetate, 2.0 mM EDTA, 0.5 mM EGTA, 10 mM Tris-HCl (pH 8.0) and 1 mM dithiothreitol (DTT)), and cells were dissociated via douncing and pipetting The resulting mixture was filtered through 100 and 70 μM strainers, respectively (Pluriselect), and the resulting pellet was resuspended in a 0.1% PBS BSA. Cells were then incubated with cardiac troponin T conjugated with 488 fluorophores (dilution=1:100) for 20 minutes. After 20-minute incubations, cells were spun down at 750g for 10 minutes. The resulting cell pellet was resuspended in 1 mL of 0.1% BSA in PBS for sorting.

### Human induced pluripotent stem cell culture and cardiomyocyte differentiation

WTC-11 which is a wild-type human male iPSC line (Coriell Institute: # GM25256), and WTC-Cas9 (Control) that was derived from WTC-11 by inserting CAG-rtTA::TetO-Cas9 (Addgene #73500) into the AAVS1 locus to yield a dox-inducible Cas9 hiPSC line was used in the study. Media preparation followed standard supplement protocols (e.g., E8, with modifications for RPMI/B27). Cells were passaged at 90% confluency using Versene and Y-27632 and cryopreserved in mFreSR. Differentiation was achieved using a stepwise protocol involving CHIR909921 and IWR1-endo. The details of the preparation steps and materials followed have been described previously^63^.

### Flow cytometry to sort diploid cardiomyocyte

To accurately identify cardiomyocyte nuclei, single cardiac nuclei were isolated and analyzed using FACS-based cardiac troponin T (cTnT) staining, nuclear DAPI intensity, and a FACSAria (20 psi, 100-mm nozzle, Becton Dickenson Biosciences) machine. A gating strategy was used to select large, granular cells with high forward and side scatter values, and to discriminate against cell doublets. Cell doublet discrimination was performed by a combination of high forward scatter height and area FSC-H/FSC-A and SSC-H versus SSC-W plots. H versus W or A allows separating the doublets from the single cells. Cells that were CnTn^+^ were sorted as single cells in 96-well plates. The genomes of single diploid cardiomyocytes were amplified using PTA^16^.

### Whole-genome amplification using PTA

Single cardiomyocytes from control and IHD, as well as normoxic and hypoxic iPSC-CMs, were sorted into 96-well plates, and their genomes were amplified using PTA, a method that uses an isothermal DNA polymerase and a termination base to induce quasi-linear amplification. The PTA reactions were performed using the ResolveDNA Whole Genome Amplification Kit. The cells were sorted into 3 microliters of pre-chilled Cell Buffer, lysed with the addition of 3 microliters of MS Mix, and then neutralized with 3 microliters of SN1 buffer. After adding 3 microliters of SDX reagent and incubating for 10 minutes at room temperature, 8 microliters of reaction mix containing enzyme were added, resulting in a total reaction volume of 20 microliters. The amplification was carried out for 10 hours at 30°C, followed by enzyme inactivation at 65°C for 3 minutes. The amplified DNA was cleaned up using AMPure, and the yield was determined using PicoGreen (Quant-iT dsDNA Assay Kit, Thermo Fisher Scientific) binding. The amplified genomes were subjected to quality control using multiplex PCR and Bioanalyzer, and those showing positive amplification for all four multiplex PCR loci were prepared for Illumina sequencing. The DNA was sequenced on the Illumina NovaSeq platform at a coverage of 30X. Libraries were prepared using a modified protocol based on the KAPA HyperPlus Library Preparation method and the ResolveDNA EA Whole Genome Amplification protocol. The process included end repair and A-tailing of 500 nanograms of amplified DNA input, adapter ligation using the SeqCap Adapter Kit, and PCR amplification of the ligated DNA. The amplified libraries were selected to have a size between 300 and 600 base pairs using AMPure and were subjected to quality control using PicoGreen and the Tape Station HS DS100 Screen Tape. The single-cell genome libraries were sequenced on the Illumina NovaSeq platform at a coverage of 30 times (30X).

### Read alignment and generation of BAM files

Reads generated from WGS were mapped onto the human reference genome (GRCh37 with decoy) by BWA (v0.7.15) with default parameters. Duplicate reads were marked by MarkDuplicates of Picard tools (v2.8) and post-processed with local realignment around indels and base quality score recalibration using Genome Analysis Toolkit (GATK) (v3.5).

### Determine sequencing quality metrics and evenness of whole-genome amplification

The sequencing quality metrics, including depth and coverage, were calculated using samtools (v1.15.1). The evenness of single-cell genome amplification was assessed using two metrics: the median absolute pairwise difference (MAPD) and coefficient of variation (CoV). The MAPD metric, as described previously^64^, quantifies amplification unevenness by calculating the median of all absolute differences between the log2-transformed copy number ratios of adjacent genomic bins. Higher MAPD scores indicate greater amplification unevenness. Similarly, the CoV was calculated by dividing the standard deviation of the absolute differences between neighboring bins by their mean. Higher CoV values also reflect greater amplification variability (Supplementary Table 1).

### Somatic mutation calling with SCAN2

The analysis was performed using SCAN2 (v1.1; commit ID ac231ce) and the associated R package r-scan2 (commit ID aa3d90e). The mutation calling was conducted in two batches, corresponding to the two sets of delivered data. The total number of samples that passed QC is shown in Fig. 1c (50 cardiomyocytes from ten controls and 33 cardiomyocytes from five IHD cases). To begin with the analysis of the first data set, a cross-sample panel required for indel calling with SCAN2 was constructed using all 33 BAMs (18 control and 15 IHD cardiomyocytes) across 13 individuals. The configuration was carried out via the SCAN2 config command with the --analysis makepanel parameter, employing the GRCh37 human reference genome with the hs37d5 decoy (--ref), dbSNP v138 (--dbsnp), and the 1000 Genomes Phase 3 SHAPEIT2 phasing panel (--shapeit-refpanel), as described in Luquette et al.^17^. For each of the 33 BAM files, a corresponding --bam argument was supplied, along with a metadata file using --makepanel-metadata to map each sample ID to an individual ID. The panel was then generated via the SCAN2 makepanel command.

Subsequently, the SCAN2 config command was executed in --analysis=call_mutations mode for each individual separately, using the same GRCh37 reference, dbSNP, and phasing panels as in the previous step. All single-cell PTA data and bulk files from the individual were included via --sc-bam (for PTA cells) or --bulk-bam (for matched bulk). The previously generated cross-sample panel was provided via --cross-sample-panel. After configuration, mutation calling was performed using the scan2 run command, resulting in an R object (scan2_object.rda) containing the raw SNV and indel calls derived from GATK results. Mutation calling for the second data set, which included 50 cardiomyocytes (30 control and 20 IHD) that passed QC out of 59 cardiomyocytes, was performed using the same approach.

Since SCAN2’s somatic calling sensitivity was influenced by sequencing depth, a trimmed mean approach was implemented to calculate the estimated mutation burden, designed to address depth-driven sensitivity variations (see R package r-scan2 script mutburden.R, https://github.com/parklab/r-scan2/tree/main/R). The approach worked well for samples with sufficient sequencing depth and coverage. However, for samples with lower depth and coverage, SCAN2 detected a typical number of raw SNV calls, but the trimmed mean approach resulted in NA values for the estimated burden. To address this issue, we refined the sensitivity estimation by excluding the genome regions with zero depth and then applying the usual calculation to the remaining data. This modification enabled us to recover a reasonable mutation burden estimate for these samples, which was comparable to other single cells from the same individual (see Extended Data Fig. 1e). Following this refinement, we defined an integrated QC score, QC_score = 0.3 × Depth / max(Depth) + 0.3 × Coverage / max(Coverage) + 0.2 × (1 − MAPD / max(MAPD)) + 0.2 × (1 − CoV / max(CoV)), to filter out cardiomyocytes that did not meet the quality threshold. A total of 83 cardiomyocytes with QC_score > 0.5 were included in the mutational burden analysis (Fig. 1d–f). The rest of all subsequent analyses were performed using the first set of 33 cardiomyocytes (Supplementary Table 1).

### Base substitution type and strand bias analysis

The total sSNVs were categorized into six types of point mutations, with a distinction between C>T at CpG sites and other sites (Fig. 2a and b). Mutations in transcribed regions of the genome can exhibit strand bias, characterized by differing mutation densities between the transcribed and untranscribed strands. This bias may arise from asymmetric mutagenesis and/or differential DNA repair activity between the strands. The transcriptional strands of genic sSNVs were assigned using UCSC TxDb annotations via MutationalPatterns (v3.12.0)^65^. Base substitutions (‘C’ or ‘T’) on the same strand as the gene definition were classified as “untranscribed”, whereas substitutions on the opposite strand were classified as “transcribed”. Strand bias analysis was conducted on sSNV in both control and IHD cardiomyocytes, with statistical significance determined using the Wilcoxon rank-sum test (Fig. 2c).

### Linear mixed-effects modeling of mutation burden

To investigate the association between somatic mutations and key covariates such as including age and disease conditions, we applied a continuous linear mixed-effects regression model using the lme4 R package (v1.1–35.3)^66^. The models were fitted using the maximum likelihood method, and P values for fixed effect were computed with the lmerTest R package (v3.1–3)^67^, which utilizes a t-test based on the Satterthwaite approximation.

Firstly, to estimate the age-related accumulation rate for sSNV burden in control cardiomyocytes, we developed the model (1): $y_{ij}=\beta_{0}+\beta_{1}\times \alpha_{j}+u_{j}+\epsilon_{ij}$, where $y_{ij}$ represents the sSNV burden in cardiomyocytes i from the individual j, $\beta_{0}$ estimates the number of sSNV at birth, $\beta_{1}$ quantifies the fixedeffect of age, $\alpha_{j}$ is the age of the individual j, $u_{j}$ denotes the random batch effect for individual j, assumed to follow a Gaussian distribution $u_{j}\sim N\left(0,{\sigma_{u}}^{2}\right)$, $\epsilon_{ij}$ represents the residual measurement error for cardiomyocytes i in individual j, assumed to follow a Gaussian distribution $\epsilon_{ij}\sim N\left(0,\sigma^{2}\right)$ (Fig. 1d).

Secondly, to evaluate the variation in sSNV burden between IHD and control cardiomyocytes while controlling for age, we constructed the model (2): $y_{ij}=\beta_{0}+\beta_{1}\times \alpha_{j}+\beta_{I}+u_{j}+\epsilon_{ij}$, where $\beta_{I}$ is the fixed effect of the disease condition (IHD versus control), and $y_{ij}$, $\beta_{0}$, $\beta_{1}$, $\alpha_{j}$, $u_{j}$, and $\epsilon_{ij}$ are defined as previously (Fig. 1e). This analysis revealed that IHD significantly increases the sSNV burden in cardiomyocytes. To address potential confounding effects of sequencing depth, coverage, and genome amplification evenness, we extended the model by incorporating an additional covariate, $\eta_{ij}$, representing these technical variables, including amplification metrics like MAPD and CoV. The difference in $\eta_{ij}$-adjusted sSNV burden between IHD and control cardiomyocytes remained significant (Extended Data Fig. 1a–d). The Cov, MAPD, coverage, and depth adjusted sSNVs all showed a clear aging trend with rate 7.91 (P = 0.018), 7.23 (P = 0.031), 7.69 (P = 0.022), and 8.10 (P = 0.015) sSNVs per GB per year, and a significant excess of 669.81 (P = 0.046), 740.80 (P = 0.030), 706.46 (P = 0.040), and 863.40 (P = 0.023) sSNVs per GB in IHD compared to controls (linear mixed-effects model). Beyond the genome-wide sSNV burden, we also analyzed signature-specific sSNV burden with similar models (Fig. 2e–j, Extended Data Fig. 2 b–e, g–i). When the total signature-specific sSNV burden in control cardiomyocytes is extremely low (e.g., in our case, SBS44 and SBS92 exhibit fewer than 6 sSNVs per GB across all 18 control cardiomyocytes), model (1) failed to produce a P-value. To resolve this singularity issue, we excluded the random batch effect $\left(u_{j}\right)$ and simplified the model to a linear regression (model 3): $y_{ij}=\beta_{0}+\beta_{1}\times \alpha_{j}+\epsilon_{ij}$, where $y_{ij}$, $\beta_{0}$, $\beta_{1}$, $\alpha_{j}$, and $\epsilon_{ij}$ retain the same definitions. Notably, SBS5 exhibited a distinct age-related trend line among the control samples (4.37 sSNVs per GB per year, P = 4.10 × 10^−10^, Fig. 2e, blue solid line) but showed no significant increase in IHD (P = 0.98). Similarly, SBS1 demonstrated a clear age-related accumulation trend (0.45 sSNVs per GB per year, P = 2.10 × 10^−4^, Extended Data Fig. 2g, blue solid line) but did not show a significant increase in IHD (P = 0.11). Nonetheless, the linear mixed-effects model detected significant elevations in several other mutational signatures in IHD cardiomyocytes. SBS8 exhibited no significant aging effects (P = 0.76) but showed a significant increase in IHD (14.61 sSNVs per GB, P = 0.042, Fig. 2f). Similarly, SBS30 showed no significant aging effects (P = 0.060) but a significant increase in IHD (61.41 sSNVs per GB, P = 0.030, Fig. 2g). SBS32 demonstrated no significant aging effects (P = 0.20) but a significant increase in IHD (35.53 sSNVs per GB, P = 0.034, Fig. 2h). SBS44 also showed no significant aging effects (P = 0.31), but significant increase in IHD (30.77 sSNVs per GB, P = 0.028, Fig. 2i). Lastly, SBS89 exhibited no significant aging effects (P = 0.20) but a significant increase in IHD (21.56 sSNVs per GB, P = 0.026, Fig. 2j). We observed an insignificant contribution of the mutational burden from SBS4 and 92 in IHD cardiomyocytes (Extended Data Fig. 2h–i). IHD donors also had a history of cigarette smoking, and SBS4 and 92-related mutations have been associated with tobacco smoking. We hypothesize that the origin of SBS4 and SBS92-related mutations in IHD cardiomyocytes could be from cigarette smoking. It is significant that we found a strong smoking signature in myocardial cardiomyocytes, which are only exposed to tobacco toxins through the blood. Due to limitations in the current version of SigNet, we could not retrain the neural network with the four *de novo* signatures (N1, N2, N3, and N4). Instead, we used their contributions, as generated by the MutationPatterns package, for the burden analysis. The linear mixed-effects model identifies that signature N1 demonstrated a clear age-related accumulation trend (2.82 sSNVs per GB per year, P = 8.10 × 10^–10^, Extended Data Fig. 2b) but showed no significant increase in IHD (P = 0.63). Signature N2 showed neither a significant age-related accumulation trend (P = 0.12) nor a significant increase in IHD (P = 0.054, Extended Data Fig. 2c). Signature N3 demonstrated a clear age-related accumulation trend (1.45 sSNVs per GB per year, P = 0.012, Extended Data Fig. 2d) but showed no significant increase in IHD (P = 0.25). Signature N4 demonstrated a clear age-related accumulation trend (0.18 sSNVs per GB per year, P = 0.037) and a significant increase in IHD (188.39 sSNVs per GB, P = 0.036, Extended Data Fig. 2e). Additionally, we included gender as a covariate in the model and confirmed that gender does not have a significant effect on the sSNV burden.

### Mutational signature analysis

The mutational signatures of sSNVs, combining both control and IHD cardiomyocytes, were derived from the 96-trinucleotide contexts somatic mutational spectra. The aggregated spectra for control, IHD, and their net differences (IHD spectrum minus control spectrum) are presented in Fig. 2b. To achieve a comprehensive understanding of the mutational signatures, we implemented two approaches. Firstly, we decomposed the overall mutational spectra (aggregated across all control and IHD cardiomyocytes) into COSMIC mutational signatures^68^ using a deep-learning method named SigNet^53^. The deep neural network (NN) was retrained with 55 COSMIC signatures (v3.3.1), excluding potential artifacts signatures (see Supplementary Table 10; blank entries were due to NN training issues in the current version of SigNet). The optimal contributions of COSMIC signatures to the overall mutational spectra are shown in Fig. 2d. As anticipated, SBS5 was a dominant contributor in both control and IHD samples, alongside signatures associated with oxidative damage (SBS8) and DNA repair pathway deficiencies (SBS30 and SBS44).

Secondly, we performed de novo extraction of mutational signatures and calculated their contributions to the total mutational spectrum using non-negative matrix factorization (NMF), as implemented in MutationalPatterns (v3.12.0). Four *de novo* signatures (N1, N2, N3, and N4) were identified based on two criteria: the cophenetic correlation coefficient (determining the point at which it begins to decrease) and the residual sum of squares (identifying the inflection point)^69^ (Extended Data Fig. 2a). To investigate the potential underlying mechanisms of these de novo mutational signatures, we used SigNet to further decompose them into COSMIC signatures (Extended Data Fig. 2f).

### Gene expression and chromatin accessibility analysis

We leverage genomic and transcriptomic data from control and IHD cardiomyocytes to investigate the potential association between somatic mutation burden and gene expression levels. We extracted cardiomyocyte expression data from our single-cell RNA-seq dataset and incorporated it with the sSNV calls. Gene expression values were normalized for each condition and averaged across all the cells using AverageExpression function in Seurat (v5.0.3). Based on the normalized expression levels, genes were classified into eight groups, with gene expression increasing from groups 1–8. The sSNV density was then calculated for each group. To minimize bias arising from trinucleotide context and the distribution of phaseable regions (which are characterized by sufficient sequencing coverage and adjacent heterozygous germline SNPs), we performed 1,000 permutations of the sSNV list for each sample. In each iteration, sSNVs were randomly shuffled within phaseable regions while preserving the original trinucleotide context distribution, using the SCAN2 permtool scripts. Finally, we calculated the permuted sSNV density for each gene expression group under control and IHD conditions, representing the expected sSNV density, and derived the observed-to-expected sSNV density ratio along with its mean and standard deviation (Fig. 3d).

Similarly, we performed the chromatin accessibility analysis to explore the relationship between somatic mutation burden and chromosome openness. The single-nucleus ATAC-seq data were preprocessed following the steps outlined in the Mutation enrichment analysis section by Ganz et al. The genome was classified into eight groups, with the chromatin accessibility levels increasing from groups 1–8, and sSNV density was calculated for each group. Then we derived the observed-to-expected sSNV density ratio as well as its mean and standard deviation for each chromatin accessibility group in control and IHD conditions (Fig. 3e).

### Annotation of genomic location and functional categories

To explore the distribution of sSNVs across genic regions and assess their potential biological impact on gene function, the sSNVs identified in age-matched control and IHD conditions were annotated and categorized by ANNOVAR (version updated in 2020June8th)^70^ separately (supplementary Table 3). Positional categories included: intergenic, upstream (within 1 kb upstream of the transcription start site), 5′ UTR, exonic (coding sequences, excluding untranslated regions), 3′ UTR, downstream (within 1 kb downstream of the transcription termination site), splicing sites (within 2 bp of an intronic splicing junction), and intronic (Fig. 3a). Functional classifications were assigned to sSNVs using four categories: synonymous (SNVs that do not alter the encoded amino acid), nonsynonymous (SNVs that result in amino acid changes, excluding stop-gain and stop-loss mutations), stop-loss (nonsynonymous SNVs that remove a stop codon), and stop-gain (nonsynonymous SNVs that introduce a stop codon), see Fig. 3b. To assess negative selection effects in cardiomyocytes sSNVs, we calculated the exonic-to-intronic ratio and nonsynonymous-to-synonymous ratio (dN/dS) for both age-matched control and IHD groups, normalizing these ratios with the corresponding germline mutation ratios. Lower exonic-to-intronic and dN/dS ratios suggested negative selection. Statistical significance was evaluated using a two-sided Fisher’s exact test. The results are shown in Fig. 3c. In the age-matched control cardiomyocytes, a marginal excess nonsynonymous sSNVs was observed compared to germline mutations (P = 0.0498), providing evidence for potential reduced negative selection constraint. In the IHD cardiomyocytes, there was a significant excess of exonic and nonsynonymous sSNVs compared to germline mutations (P = 1.50 × 10^−4^ and P = 1.15 × 10^−5^, respectively), further implying a reduction in the strength of negative selection. Circos plot showing the distribution of sSNVs across the genome for control and IHD conditions was shown in Fig. 1f.

### Single-nuclei RNA-seq analysis

snRNA-seq libraries were generated using the 10x Genomics Single Cell Gene Expression kit according to manufacturer’s instructions. Briefly, single DAPI-positive nuclei were sorted into sterile PCR tubes containing reagents as per the 10x user guide. GEMs were generated using the 10x Genomics Chromium Controller X and following the manufacturer’s protocol for 3′ V3.1 chemistry with NextGEM Chip G reagents (10x Genomics Inc., Pleasanton, CA, USA). The quality of the libraries generated after snRNA-seq protocols was assessed by running them on Tapestation 4200 (Agilent Inc., Santa Clara, CA USA). The libraries were then sent to Psomagen Inc., MD 20850 for paired-end sequencing (150 bp × 2) on a HiSeq ×10 instrument. The data from all the samples were processed systematically by doing demultiplexing on raw data. We used the 10x Genomics cellranger pipeline (v7.1.0) for snRNA-seq data pre-processing, including alignment to GRCh38 Ensemble (hg38), barcode and UMI counting, and generation of feature-barcode matrices.

We developed an in-house snRNA-seq analysis pipeline based on the Seurat R package (R v4.3.1, Seurat v5.0.3)^71^ to perform downstream analysis, including quality control, cell filtering, spectral clustering, cell type annotation, sub-clustering, differentially expressed gene (DEG), and visualization of the snRNA-seq data. DoubletFinder (v2.0.3)^72^ was used to identify and filter out doublets, employing an optimized doublet detection probability threshold (pK = 0.4), which minimizes false positives. Following QC, the average cell count per sample and gene count per nuclei were 4,759 and 1,675, respectively. To ensure data quality, we removed low-quality cells and technical artifacts based on the following criteria: cells with extremely high or low Unique Molecular Identifier (UMI) counts and numbers of unique gene features (outside the 95% confidence interval), potentially damaged or dying cells (indicated by >10% mitochondrial or >5% ribosomal gene expression), and cells with low gene diversity per UMI (log10GenesPerUMI < 0.8) (Extended Data Fig. 7). The RNA counts were normalized, and top 3000 highly variable features were selected through the vst method. Data were then scaled by regressing out the percentage of mitochondrial genes, the percentage of ribosomal genes, the total UMI count per cell (adjusts for variability in sequencing depth across cells), and the number of features detected per cell (correct for differences in library complexity or cell quality). High-quality cells were then integrated using Harmony (v1.2.0)^73^ and corrected for batch effects associated with sample and sequencing technique (3’ and 5’ protocols). Data dimension reduction was performed using the top 30 Harmony dimensions with the Uniform Manifold Approximation and Projection (UMAP)^74^, followed by clustering with FindClusters function at a resolution of 1.0. We identified the top marker genes of each unsupervised cell cluster using the FindAllMarkers function, and cell types were further characterized using well-known markers (Fig. 5a). Cell type annotation using UMAP clustering was shown in Fig. 5b. Fig. 5c presented the relative composition of different cardiac cell types for control and IHD samples. Next, cardiomyocyte and fibroblast clusters were extracted for unsupervised sub-clustering analysis, revealing 19 unique clusters across control (Fig. 5d) and IHD heart (Fig. 5e) samples, with the expression of cardiomyocyte and fibroblast marker genes shown for both (Fig. 5f and 5g). Three clusters (9, 10, and 11), which predominantly expressed cardiomyocyte markers in the control heart, shift to expressing fibroblast markers in the IHD heart (suggesting fibrotic-cardiomyocyte). The identified cardiomyocyte, fibrotic-cardiomyocyte, and fibroblast populations in control and IHD heart were shown in Fig. 5h. The relative proportion of cardiomyocytes and fibroblasts in control and ischemic heart samples are shown in Fig. 5i. IHD individuals exhibited a decrease in the proportions of cardiomyocytes and an increased in fibroblasts (P = 0.021, two-tailed Wilcoxon test). We also evaluated the average metagene (as listed in Supplementary Table 9) expression in cluster 9 and 11 from control and IHD samples, indicating downregulation of cardiomyocyte genes, upregulation of fibroblast, hypoxia, and collagen genes. Statistical significance was assessed using a two-sided Wilcoxon test (Fig. 5j). Cluster 10 was excluded from the metagene analysis as it contained very few nuclei. For DEG analysis, we extracted the cardiomyocytes clusters from sub-clustering results and excluded the individuals with fewer than 2000 cardiomyocytes, retaining 3 control individuals and 3 IHD individuals. We then applied a Leave-One-Out (LOO) approach combined with the FindMarkers function from Seurat. In each LOO iteration, one control and one IHD individual were excluded, and the FindMarkers function was executed with various combinations of logfc.threshold values for IHD up-regulated and down-regulated genes, with default Wilcoxon rank sum test and min.pct = 0.1. This method identified common DEGs across all 9 combinations of DEG groups, ensuring that DEGs were expressed in all individuals and mitigating biases from uneven individual representation in the analysis. The optimized logfc.threshold for identifying IHD upregulated and downregulated DEGs were 0.30 and 0.25, respectively (Fig. 6a–b). The IHD upregulated and downregulated genes are marked as red and blue, respectively. The complete list of upregulated and downregulated DEGs is provided in Supplementary Table 7. Pathway enrichment analysis of DEGs showed that genes related to hypoxia, inflammation, and signal transduction are upregulated in IHD cardiomyocytes, while genes involved in maintaining contractile fiber, cardiomyocyte contraction and skeletal muscle fiber development are downregulated (Fig. 6c).

### Functional enrichment analysis

To uncover the biological significance of the genomic and transcriptomic data, we performed functional enrichment analysis on genes harboring deleterious SNVs and snRNA-seq DEGs using GOseq (v1.54.0). For GO functional enrichment analysis of genes containing deleterious sSNVs, each RefSeq gene was assigned a binary value ‘0’ or ‘1’ indicating the absence or presence of deleterious sSNVs in the corresponding gene, respectively. We applied a probability weighting function in GOseq to account for potential gene-length bias (due to variations in the length of exonic regions). The full list of genes in GO terms was retrieved using AnnotationDbi (v1.64.1). GO terms with fewer than two hits or more than 1,000 genes were excluded. GO terms with P < 0.05 in either IHD or control cardiomyocytes are reported in Fig. 3f–g and Supplementary Table 5.

Similarly, for GO functional enrichment analysis of DEGs identified from snRNA-seq, each RefSeq gene was assigned a binary value ‘0’ or ‘1’ depending on its inclusion in the DEG list. A probability weighting function in GOseq was applied without gene length correction. GO terms significantly enriched (P < 0.05) in either IHD upregulated or downregulated DEGs are represented in Fig. 6c and Supplementary Table 8.

### Western blotting

To perform the western blot from human heart tissue lysis, small chunks of the tissue were washed 3X in PBS to remove any blood, followed by the tissue homogenization using homogenizer (Cole Palmer-LabGEN 125) in RIPA buffer (790 mg Tris base, 900 mg NaCl, 10 ml of 10% NP-40, 2.5 ml of 10% Na-deoxycholate, 1 ml of 100 mM EDTA, distilled H2O). The homogenized tissue was kept in an ice-cold lysis buffer for 45 minutes with 15s vortexing every 10 minutes. To perform the cell lysis from iPSC CM, cells were washed with PBS and lysed with the same RIPA buffer under the same conditions. After 45 minutes, the lysate was centrifuged at 14,000g for 30 minutes and the supernatant was transferred to fresh tubes. Total protein concentration in the lysates was determined using a BCA Protein Assay Kit (Thermo Fisher Scientific Inc.). Protein lysates were subjected to SDS-PAGE and then transferred to a polyvinylidene difluoride membrane (PVDF).

PVDF membrane was blocked using 5% milk in 1% PBST (Bio-Rad) for 1Hr at room temperature, the membranes were probed with MSH2 (Cell Signaling Technology #9234, 1:1000 dilution), MSH6 (Cell Signaling Technology #4058, 1:500 dilution), MLH1 (Cell Signaling Technology #3515, 1:1000), MLH3(Ab Clonal Cat no. A25504, 1:1000), DNA-PKs(Cell Signaling Technology #38168, 1:1000), XRCC1(Cell Signaling Technology #2735, 1:1000), DNA ligase IV (Cell Signaling Technology, #14649, 1:1000 dilution) at 4 °C overnight. Membranes were then washed with PBST 3 times for 10 minutes each (GIBCO) and incubated with the secondary antibody to Anti-rabbit and Anti-mouse IgG HRP-linked Antibody (Cell Signaling Technology #7074, 1:10,000) at room temperature for 1 hour. After washing in PBST, the immunoblots were visualized using ECL detection reagents (NACALAI TESQUE, INC. #02230–30). ß-Actin (Cell Signaling Technology #4967, 1:10,000) was used as a loading control. The area and intensity of the image files were measured using ImageJ, and the signal was calculated by integration.

### Immunofluorescent staining on frozen heart tissue and iPSC-CMs

Fresh-frozen left ventricular human heart tissue was embedded in OCT and cryosectioned (10–12μ). Tissue slides were stored at −80°C until further use. Tissue sections were fixed with 4% PFA and permeabilized for 15 minutes. Differentiated cardiomyocytes were washed with PBS 3- times and fixed with 4% paraformaldehyde (PFA) for 20 minutes. The cells were then rinsed 3 times for 5 minutes each. The cells were permeabilized with 0.05% Triton-X in PBST supplemented with 1% donkey serum for 10 minutes twice. Permeabilized tissue section and cells were blocked using 5% donkey serum in PBS for 30 minutes. The tissue and cells were then stained for markers for DNA damage, chromatin dysregulation, and hypoxia, including H2Ax **(**Cell Signaling Technology #2577, 1:1000**)** and 8-oxoG (Abcam, Ab2Q2311, 1:1000), overnight at 4°C. Tissue sections and cells were washed four times with 1% donkey serum in PBS–Tween 20 (DPBS-T), for 10 minutes each time, and then incubated for 1 hour at RT in the dark with Alexa-Fluor-conjugated secondary antibodies (Life Technologies) (1:400, diluted in 2% donkey serum). Tissue sections and cells were washed again as described above, mounted with DAPI mounting solution (Life Technologies) onto Superfrost Plus (Thermo Scientific) slides, and imaged with and confocal microscope (Zeiss).

### Picro Sirius Red staining:

Immunostaining was performed using a standard protocol. Briefly, fresh-frozen left ventricular human heart tissue was embedded in OCT and cryosectioned (10–12μ). Tissue slides were stored at −80°C until further use. Tissue sections were air-dried for 30 minutes, then processed through a graded ethanol series (100%, 80%, and 40%), followed by a wash in distilled water. Slides were fixed in formalin for 30 minutes and washed again with water. Sections were stained with Picro Sirius Red (Catalog #AB246832) for 1 hour at room temperature and washed in two changes of 0.5% acetic acid for 2 minutes each to differentiate collagen from nonspecific background staining. Slides were then quickly dehydrated through graded ethanol (40%, 80%, and 100%) and cleared with two changes of xylene before mounting coverslips with a suitable mounting medium. This method effectively highlights collagen fibers for fibrosis detection.

## Extended Data

## Data Availability

scWGS data will be deposited in the NCBI dbGaP with an accession number. The data are available under controlled use conditions set by human privacy regulations. Other data are available upon request.
